# Effects of acupuncture at acupoints with lower versus higher pain threshold for knee osteoarthritis: a multicenter randomized controlled trial

**DOI:** 10.1186/s13020-022-00626-3

**Published:** 2022-06-08

**Authors:** Jiali Liu, Ying Li, Ling Li, Xiaochao Luo, Ning Li, Xuguang Yang, Hongxing Zhang, Zhibin Liu, Deying Kang, Yanan Luo, Yanmei Liu, Yulong Jia, Yan Ren, Minghong Yao, Yuning Wang, Jin Chen, Mewujia Maiji, Kang Zou, Ling Zhao, Fanrong Liang, Xin Sun

**Affiliations:** 1 Chinese Evidence-Based Medicine Center and Cochrane China Center, West China Hospital, Sichuan University, 37 Guo Xue Xiang, Chengdu, 610041 Sichuan China; 2 NMPA Key Laboratory for Real World Data Research and Evaluation in Hainan, Chengdu, 610041 Sichuan China; 3 Sichuan Center of Technology Innovation for Real World Data, Chengdu, 610041 Sichuan China; 4 Graduate School, Chengdu University of Traditional Chinese Medicine, Chengdu, 610075 Sichuan China; 5 Department of Integrated Traditional Chinese and Western Medicine, West China Hospital, Sichuan University, Chengdu, 610041 China; 6 College of Acupuncture and Massage, Henan University of Traditional Chinese Medicine, Zhengzhou, 450008 China; 7 Department of Acupuncture, Wuhan Hospital of Traditional Chinese and Western Medicine, Wuhan, 430022 China; 8 Acupuncture and Tuina School, Chengdu University of Traditional Chinese Medicine, Chengdu, 610075 Sichuan China; 9 Evidence-Based Medicine Research Center, School of Basic Science, Jiangxi University of Traditional Chinese Medicine, Nanchang, 330004 Jiangxi China

## Abstract

**Background:**

The acupoint selections impact the effects of acupuncture, and preliminary evidence showed potential connection between pain threshold (PT) and acupuncture response. This study examined whether acupuncture at acupoints with lower PT versus higher PT would yield different effects in patients with knee osteoarthritis (KOA).

**Methods:**

In this multicenter randomized clinical trial, patients were randomly assigned (1:1:1) to receive acupuncture at acupoints with lower PT (LPT group), acupuncture at acupoints with higher PT (HPT group), and no acupuncture (waiting-list group). PT was measured with electronic von Frey detector. The primary outcome was the change in WOMAC total score from baseline to 16 weeks, and the secondary outcomes were SF-12 score, and active knee range of motion (ROM). Intention-to-treat analysis was conducted with linear mixed-effect model.

**Results:**

Among 666 randomized patients, 625 (93.84%) completed the study. From baseline to 16 weeks, patients in the LPT group versus HPT group had similar effects in reducing WOMAC total score (adjusted mean difference (MD) 2.21, 95% confidence interval (CI) −2.51 to 6.92, P = 0.36), while a greater reduction in WOMAC total score was observed in LPT group (−9.77, 95% CI −14.47 to −5.07, *P* < 0.001) and HPT group (−11.97, 95% CI −16.71 to −7.24, *P* < 0.001) compared with waiting-list group. There were no differences in SF-12 score and knee ROM between LPT versus HPT groups.

**Conclusion:**

Our findings found that the effects of acupuncture at acupoints with lower versus higher PT were similar, both were effective for patients with KOA.

*Trial registration*: ClinicalTrials.gov identifier: NCT03299439. Registered 3 October 2017, https://clinicaltrials.gov/ct2/show/NCT03299439

**Supplementary Information:**

The online version contains supplementary material available at 10.1186/s13020-022-00626-3.

## Introduction

Worldwide, knee osteoarthritis (KOA), characterized by gradual loss of joint cartilage and local inflammatory processes, is the fastest growing health disorder and the most common cause of disability [1, 2], and has resulted in considerable socioeconomic burdens [3]. The management of KOA usually starts with non-drug treatment (e.g., education and exercise) and medications (e.g., nonsteroidal anti-inflammatory drugs, NSAIDs), and ends up with joint replacement surgery [4–6]. Due to undesirable side effects of long-term pharmacological treatments and knee surgery [7–9], complementary and alternative medical (CAM) therapies are increasingly used [10]. Acupuncture treatment represents the most popular CAM therapy [11, 12], evidence from clinical trials and systemic reviews has suggested that acupuncture can be effective in treating pain and dysfunction in patients with KOA [9, 13–15].

Selection of acupoints is one of the determinative factors influencing acupuncture effect, as it is the first step and foundation of acupuncture operation [16, 17]. In the current clinical practice, the composition of acupoint prescriptions is mainly based on acupuncturists’ clinical experience, and the choice of acupoints varies depending on the acupuncturists’ practice style [18, 19]. This subjective and unquantifiable pattern may be difficult for replicating or evaluating acupuncture effect, which limits the application of acupuncture in clinical practices [20]. The pain threshold (PT) is a valid and reliable measure of quantifiable localized pain [21]. Studies have suggested that acupuncture can achieve the analgesia effect by stimulating somatic sensory functions of nervous system, further activating the endogenous pain inhibitory systems (e.g., inhibition of the nociceptive pathway at the dorsal horn by activation of the descending inhibitory pathways), and finally increasing the PT of pain sites in patients with chronic musculoskeletal pain [22–24]. Furthermore, a recent trial showed that fibromyalgia patients with lower or higher baseline PT had differential treatment response to acupuncture [25], suggesting a potential connection between PT of local pain sites and acupuncture effects in patients with chronic pain. Given KOA is mainly manifested as chronic pain, this raises a biologically plausible query as to whether the effects of acupuncture would vary by acupoints with different PT in patients with KOA.

We thus conducted this 16-week randomized clinical trial (RCT) to examine whether acupuncture at lower PT versus higher PT acupoints around knee would result in different treatment response in patients with KOA.

## Methods

### Study design

This was a three-arm, parallel, and multicenter RCT conducted at four teaching hospitals (i.e., Affiliated Hospital of Chengdu University of Traditional Chinese Medicine, West China Hospital of Sichuan University, Third Affiliated Hospital of Henan University of Traditional Chinese Medicine, and Wuhan Integrated Traditional Chinese Medicine and Western Medicine Hospital) in China between October 2017 and November 2020. Eligible patients were recruited from the outpatient departments of Acupuncture and Moxibustion, Integrated Chinese-Western Medicine, and Rehabilitation Medicine. This trial was approved by the ethics review board of the Bioethics Subcommittee of West China Hospital, Sichuan University (Approval No. 228), registered at ClinicalTrials.gov (No. NCT03299439), and overseen by an independent data monitoring committee (DMC). We conducted a pilot study to inform the design and feasibility of the current trial [26], suggesting the need to include a waiting-list control group. We thus used a three-arm design to help evaluate assay sensitivity of this trial [27]. The trial protocol was previously published [28]. We followed the CONSORT and STRICTA guidelines to report this study [29].

### Patients

Patients were included if they were 40 years or older and diagnosed with mild or moderate KOA (Grade 0-III, according to Kellgren-Lawrence criteria). The diagnostic criteria were followed according to the Chinese Guideline for the Medical Management of KOA [30].

Patients with any of the following conditions were excluded: diagnosed with tuberculosis, tumors, rheumatism of the knee joint, and rheumatoid arthritis; sprain or trauma in the lower limb; present with mental disorders; present with comorbidities that severe cardiovascular disease, liver or kidney impairment, immunodeficiency, diabetes mellitus, blood disorder or skin disease; pregnancy or lactation; use of physiotherapy for osteoarthritis knee pain in the past month; use of intra-articular injection of glucocorticoid or viscosupplementation in the past 6 months; received knee-replacement surgery; and positive floating patella test.

Patients have the right to withdraw from the trial at any time. Investigators also have the right to require the patient to suspend the trial for medical reasons such as serious adverse events in the interest of patient.

### Randomization and blinding

Patients were randomly allocated, at a ratio of 1:1:1, into a lower PT group (LPT group, that is, acupuncture at acupoints with lower PT), a higher PT group (HPT group, that is, acupuncture acupoints with higher PT) or a waiting-list group (no acupuncture). The randomization was conducted via a central randomization system, and the randomization sequence was generated in a block size of 3 or 6 and stratified by participating sites. Assignment of patients was performed thorough the central system by an independent coordinator. Patients in LPT and HPT groups were blinded to allocation (patients in LPT group and HPT group were informed to receiving the same acupuncture treatment), and were required not to release their treatment information to outcome assessors during the study. Outcome assessors and data analysts were blinded to treatment allocation.

### Identification, measurement and selection of acupoints

#### Identification of acupoints

We identified 13 acupoints around knee joint for treating KOA according to literature and expert consensus [31]. The acupoints included Heding (EX-LE2), Neixiyan (EX-LE4), Dubi (ST35), Xuehai (SP10), Liangqiu (ST34), Yinlingquan (SP9), Yanglingquan (GB34), Zusanli (ST36), Weizhong (BL40), Yingu (KI10), Xiguan (LR7), Ququan (LR8) and Weiyang (BL39). In addition, we also identified *ashi* point from 12 testing areas around knee based on anatomical structure and expert consensus (Additional file 1: Figure S1).

#### Measurement of PT of acupoints

Trained acupuncturists measured PT with the electronic von Frey detector (2390 series, IITC Life Science). Each point was tested twice at an interval of 2 min. If the difference between the two values was greater than 15 g/N, a third measure was made at this point. The average of two values with the smallest difference was recorded as the final PT of the tested acupoint.

#### Selection of acupoints for interventions

The acupoints were ranked by the PT value. The five points with lowest PT were identified as lower PT acupoints (corresponding to LPT group), and the five with the highest PT as higher PT acupoints (corresponding to HPT group).

#### Interventions

Sterile, single-use needles (Hwato Needles, Sino-foreign Joint Venture Suzhou Hwato Medical Instruments, China) with a length of 40 mm and a diameter of 0.30 mm were used. Acupuncture was performed by acupuncturists who did not participate in identification and measurement of acupoints. The acupuncturists were specialists in Traditional Chinese Medicine at the hospitals, received specialized acupuncture training and licensed with at least 3 years of clinical experience.

In the LPT group, patients received acupuncture treatment at five lower PT acupoints. After skin disinfection, the needles were inserted vertically into the acupoints with a depth of 15–30 mm. The stimulation was performed with lifting and thrusting combined with rotating to induce the sensation known as *de qi* (sensation of soreness, numbness, distention, or radiating) [32]. Patients received 12 sessions of acupuncture (three sessions per week or every other day) for four consecutive weeks. Each session lasted 30 min and acupuncture needle manipulation was performed every 15 min.

In the HPT group, patients received acupuncture at five higher PT acupoints, and all the other treatment settings were the same with those in the LPT group.

In the LPT and HPT groups, patients with unilateral KOA were treated with acupuncture on the affected side. Patients with bilateral KOA were treated and assessed on their most painful side, and the non-trial affected low limbs were provided with acupuncture treatment on ST35, EX-LE4, GB34, ST36 and SP10. Patients in the waiting-list group did not receive any acupuncture from the beginning of the trial but were informed that they would be offered with 12 sessions of acupuncture treatment for free after the study.

All the patients were advised not to use any other treatments for KOA. However, if the patient had intolerable pain and the outcome assessment was not scheduled within the next 48 h, NSAIDs were allowed. For patients in the waiting-list group, non-acupuncture treatments, such as application of medicinal liquor on the knee, massage and moxibustion, were allowed if they requested treatment. All the above treatments were recorded, including the name, dosage, and duration of treatment.

#### Outcome measures

The primary outcome was the change of Western Ontario and McMaster Universities Osteoarthritis Index (WOMAC) total score (a composite total score of pain, stiffness, and physical function) from baseline to 16 weeks. The WOMAC, a disease-specific scale with high reliability and validity, has been translated into different languages and used widely in clinical trials for KOA. The Chinese version of WOMAC contains 24 items that measure pain (5 items, scored 0–50), stiffness (2 items, scored 0–20) and physical function (17 items, scored 0–170), with a total score ranging from 0 to 240 [33]. Each WOMAC item is rated on a Visual Analogue Scale (VAS) of 0 to 10, with a high score indicating a worse symptom.

The secondary outcome included the change of WOMAC subscale score (pain, stiffness and physical function), 12-item Short Form Health Survey (SF-12), active knee ranges of motion (ROM) and adverse events. The validated Chinese version of SF-12 consists of eight domains and may generate two separate summary scores including physical component score (PCS) and mental component score (MCS) [34], this self-report questionnaire measures quality of life. The active ROM were assessed by using a standard goniometer, including flexion, extension, internal rotation and external rotation. Any adverse events, especially the acupuncture-related adverse events that including bleeding, subcutaneous hemorrhage, hematoma, fainting, bruising, soreness and local infection at needle sites, were documented and followed up during the study.

All outcome measures were performed at baseline, 4, 8, 12 and 16 weeks. The outcome assessments, following a standard protocol [28], were performed in a separate room at the outpatient department of the research site by trained nurses, who were blinded to treatment allocation.

#### Sample size estimation

The sample size calculation was based on the mean difference of WOMAC total score changes from baseline to 16 weeks according to the pilot trial [26]. A sample size 189 patients per group was required to achieve 90% power to detect a mean difference of WOMAC total score of 12 between LPT group and HPT group when the standard deviation (SD) was 33, and the significance level was 0.025 (adjusted for multiple testing) for a two-sided test. This sample size was sufficient to detect the difference between LPT group and waiting-list group, given that the treatment effect between LPT group and HPT group would be smaller than that between LPT group and waiting-list group. The sample size of 222 individuals per group (total of 666) was allowed considering 15% loss to follow up.

#### Monitoring

An independent data monitoring committee (DMC), consisting of five members with epidemiologist, biostatistics, acupuncturist and specialist physicians, was developed to monitor the quality and regulatory compliance of the trial, and ensure the safety of participating patients. We developed a procedural document for the DMC meeting, and strictly followed the document. Two DMC regular meetings were held during this study.

### Statistical analysis

Baseline characteristics and clinical outcomes were summarized using means and SDs for continuous variables, and counts and percentages for categorical variables. The primary outcome was analyzed according to the intent-to-treat principle (i.e., full analysis set), which included all patients randomized. Missing data on primary outcome were multiply imputed using chained equations with predictive mean matching under missing at random assumption, and the estimates from 200 imputed datasets were combined by rubin rules. The change from baseline in WOMAC total score over time was analyzed by fitting a linear mixed-effect model that included baseline value as a covariate; modeled treatment, visit and treatment × visit interaction as fixed effects; and treated sites and individuals as random effects. The same approach was used for secondary continuous outcomes.

Four prespecified subgroup analyses of Kellgren-Lawrence criteria (Grade 0, Grade I, Grade II and Grade III), BMI (< 18.5, 18.5–24 and > 24 kg/m^2^), duration of disease (< 5, 5–10 and > 10 years), and unilateral/bilateral KOA were conducted for the primary outcome that comparing LPT group versus HPT group by adding interaction term (i.e., BMI × group) into the linear mixed-effect model.

A sensitivity analysis was performed for the primary outcome basing on the per-protocol set, which included patients who adhere to the treatment sessions and follow ups. A post hoc analysis of the comparison of HPT versus WL group was performed in the full analysis set and per-protocol set. All analyses were implemented using R software (version 3.6.3).

## Results

**Fig. 1:**
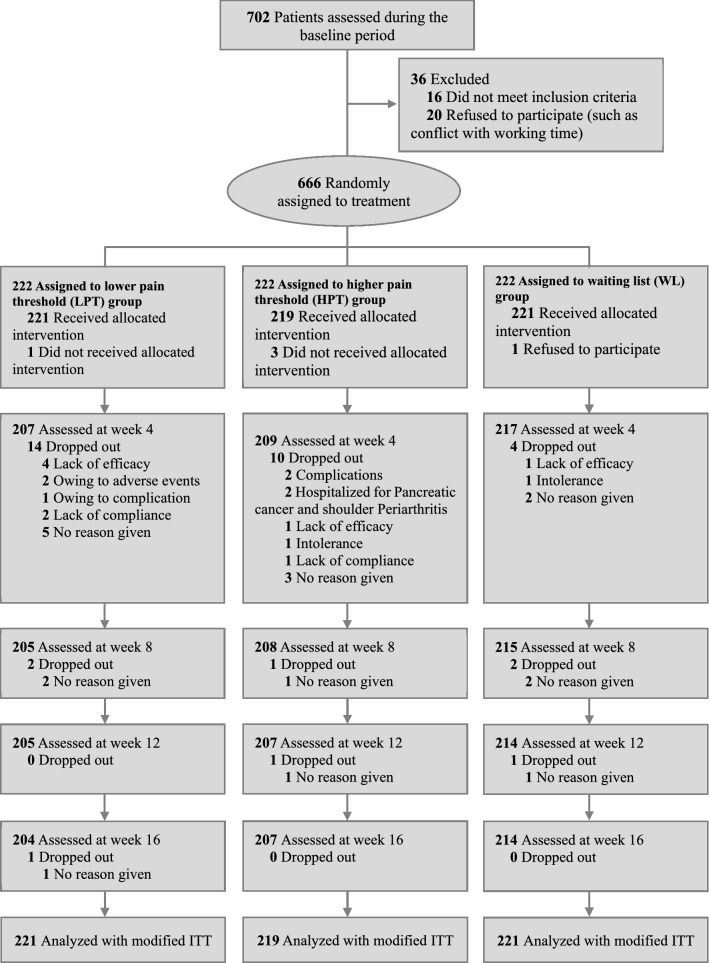
Study participant flow diagram

A total of 702 patients with KOA were screened for eligibility, of whom 36 were ineligible or refused to participate, and 666 were finally enrolled and randomized (222 at each group) (Fig. 1). A total of 625 patients (93.84%) completed the study; five patients declined to participate after randomization and did not receive allocated intervention (one in LPT group, three in HPT group, and one in waiting-list group); 41 patients (6.16%) were lost to follow up (18 in LPT group, 15 in HPT group, and 8 in waiting list group; P = 0.128) for various reasons (e.g., lack of efficacy, intolerance). In total, 661 patients were included in the full analysis set and 625 in the per protocol set.

Baseline characteristics of randomized patients were balanced among the three groups (Table 1). Mean (SD) age was 60.74 (8.80) years and mean duration of KOA was 5.96 (6.95) years; 535 patients (80.3%) were diagnosed with bilateral KOA, and 53 patients (7.96%) previously used acupuncture for KOA. 406 patients (91.44%) received eight or more treatment sessions (204 in LPT group and 202 in HPT group), and 371 patients (83.55%) received 12 treatment sessions (184 in LPT group and 187 in HPT group). Thirteen patients (1.95%) used NSAIDs during the trial, including five in the LPT group, three in the HPT group and five in the waiting-list group. Other co-interventions were similar between treatment groups (Additional file 2: Table S2).

**Table 1:** Baseline characteristics of patients

Characteristic	LPT group (n = 222)	HPT group (n = 222)	WL group (n = 222)	All (n = 666)
Age, mean (SD), y	60.46 (9.10)	61.35 (8.73)	60.41 (8.56)	60.74 (8.80)
Sex (female), n (%)	174 (78.38)	176 (79.28)	185 (83.33)	535 (80.33)
BMI, mean (SD)	24.18 (3.19)	23.91 (2.89)	24.06 (2.94)	24.05 (3.00)
Duration of disease, mean (SD), y	6.19 (6.73)	6.54 (8.44)	5.14 (5.14)	5.96 (6.95)
Kellgren-Lawrence criteria, n (%)				
Grade 0	62 (28.44)	62 (28.18)	60 (27.27)	184 (27.96)
Grade I	77 (35.32)	82 (37.27)	81(36.82)	240 (36.47)
Grade II	60 (27.52)	59 (26.82)	61 (27.73)	180 (27.36)
Grade III	19 (8.72)	17 (7.73)	18 (8.18)	54 (8.21)
Type of KOA, n (%)				
Unilateral	38 (17.12)	42 (18.92)	51 (22.97)	131 (19.7)
Bilateral	184 (82.88)	180 (81.08)	171 (77.03)	535 (80.3)
Previous use of acupuncture for KOA, n (%)	21 (9.46)	14 (6.31)	18 (8.11)	53 (7.96)
WOMAC index, mean (SD)				
WOMAC total	51.84 (39.99)	55.69 (41.42)	52.4 (36.88)	53.3 (39.44)
WOMAC pain	11.57 (8.2)	12.4 (9.36)	11.35 (7.91)	11.77 (8.51)
WOMAC stiffness	4 (4.35)	3.8 (4.19)	4.02 (4.26)	3.94 (4.26)
WOMAC function	36.26 (30.01)	39.49 (31.02)	37.03 (27.15)	37.59 (29.42)
SF-12 Index, mean (SD)				
Physical health	39.22 (8.15)	38.25 (8.53)	38.64 (7.88)	38.7 (8.18)
Mental health	52.57 (10.17)	53.01 (9.59)	52.31 (10.17)	52.63 (9.97)
Active range of motion, mean (SD)				
Flexion	121.47 (14.97)	122 (12.69)	121.13 (12.59)	121.53 (13.45)
Extension	0.16 (0.67)	0.14 (0.82)	0.33 (1.18)	0.21 (0.92)
Internal rotation	26.03 (6.88)	27.38 (7.18)	26.97 (7.21)	26.79 (7.10)"
External rotation	27.71 (14.77)	27.67 (7.18)	27.56 (6.74)	27.65 (10.25)
Pressure pain threshold, mean (SD)	64.81 (41.03)	114.31 (18.00)	–	–
Acupuncture treatment received ≥ 8 sessions, n (%)	204 (91.89)	202 (90.99)	–	–
Acupuncture treatment received 12 sessions, n (%)	184 (82.88)	187 (84.23)	–	–

*LPT* lower pain threshold, *HPT* higher pain threshold, *WL* waiting-list

### Primary outcome

**Fig. 2:**
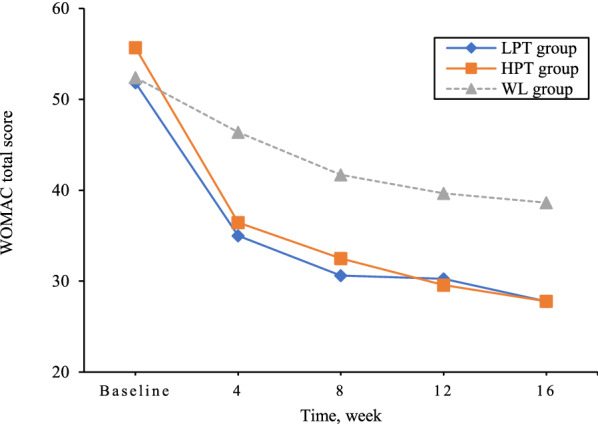
WOMAC total score over time during the study

From baseline to 16 weeks, the mean observed WOMAC total score decreased by 23.13 points in LPT group, by 27.38 points in HPT group, and by 13.51 points in waiting-list group. Patients in the LPT group versus HPT group had similar effects in reducing WOMAC total score (adjusted mean difference (MD) 2.21, 95% confidence interval (CI) −2.51 to 6.92, *P* = 0.36). A greater reduction was observed in the LPT group and the HPT group as opposed to waiting-list group (MD −9.77, 95% CI −14.47 to −5.07, *P* < 0.001; MD −11.97, 95% CI −16.71 to −7.24, *P* < 0.001) (Table 2 and Fig. 2). The per-protocol analysis showed similar results (Table 2).

**Table 2:** Comparison of primary outcome between treatment groups

Primary outcome measure	Adjusted mean difference (95% CI)^a^			Adusted model^b^					
	LPT group	HPT group	WL group	LPT group vs HPT group		LPT group vs WL group		HPT group vs WL group	
				Mean difference (95% CI)	P value	Mean difference (95% CI)	P value	Mean difference (95% CI)	P value
Change from baseline in WOMAC total score (modified intention to treat, n=661)^c^									
4 weeks	−16.49 (−19.74 to −13.25)	−19.54 (−22.99 to −16.10)	−6.20 (−9.16 to −3.24)	1.06 (−3.63 to 5.76)	0.66	−10.58 (−15.22 to −5.93)	< 0.001	−11.64 (−16.31 to −6.98)	< 0.001
8 weeks	−20.67 (−23.95 to −17.39)	−22.88 (−26.34 to −19.42)	−10.76 (−13.77 to −7.75)	0.31 (−4.43 to 5.05)	0.90	−10.11 (−14.81 to −5.41)	< 0.001	−10.42 (−15.13 to −5.71)	< 0.001
12 weeks	−20.91 (−24.23 to −17.58)	−25.41 (−28.91 to −21.91)	−12.64 (−15.66 to −9.62)	2.52 (−2.23 to 7.27)	0.30	−8.39 (−13.15 to −3.63)	< 0.001	−10.91 (−15.67 to −6.15)	< 0.001
16 weeks	−23.13 (−26.44 to −19.82)	−27.38 (−30.84 to −23.91)	−13.51 (−16.51 to −10.52)	2.21 (−2.51 to 6.92)	0.36	−9.77(−14.47 to −5.07)	< 0.001	−11.97(−16.71 to −7.24)	< 0.001
Change from baseline in WOMAC total score (per protocol, n=625)^d^									
4 weeks	−16.831 (−20.10 to −13.56)	−19.55 (−23.01 to −16.09)	−6.25 (−9.21 to −3.29)	1.09 (−3.63 to 5.82)	0.65	−10.86 (−15.54 to −6.18)	< 0.001	−11.96 (−16.62 to −7.30)	< 0.001
8 weeks	−21.52 (−24.81 to −18.24)	−23.00 (−26.47 to −19.52)	−10.73 (−13.70 to −7.76)	−0.11 (−4.84 to 4.63)	0.96	−11.14 (−15.83 to −6.44)	< 0.001	−11.03 (−15.71 to −6.36)	< 0.001
12 weeks	−21.44 (−24.73 to −18.15)	−25.84 (−29.31 to −22.36)	−12.69 (−15.66 to −9.71)	2.82 (−1.92 to 7.55)	0.24	−9.15 (−13.85 to −4.45)	< 0.001	−11.97 (−16.65 to −7.29)	< 0.001
16 weeks	−23.89 (−27.16 to -−0.62)	−27.91 (−31.37 to −24.45)	−13.68 (−16.65 to −10.72)	2.39 (−2.33 to 7.10)	0.32	−10.54 (−15.22 to −5.86)	< 0.001	−12.93 (−17.59 to −8.27)	< 0.001

*LPT* lower pain threshold, *HPT* higher pain threshold, *WL* waiting-list

^a^Adjusted analysis was performed using a liner mixed model with baseline value as covariate

^b^Adjusted analysis was performed using a liner mixed model with baseline value as covariate; treatment, visit, and treatment × visit interaction as fixed effects; sites and individuals as random effects

^**c**^All participants analyzed according to allocation (n = 661)

^**d**^All participants analyzed according to completion of 16-week follow-up (n = 625)

In subgroup analyses by Kellgren-Lawrence criteria (*P* = 0.93), BMI (*P* = 0.79), duration of disease (*P* = 0.33), and unilateral/bilateral KOA (*P* = 0.82), no apparent differences in treatments were found (Additional file 1: Figure S2).

### Secondary outcomes

No significant differences were found between LPT group and HPT group in the improvement of WOMAC pain, WOMAC stiffness, WOMAC function, SF-12 PCS, SF-12 MCS, knee flexion ROM, knee extension ROM, knee internal rotation ROM, and knee external rotation ROM at 16 weeks. The change of knee extension ROM was slightly increased in LPT group compared with HPT group (MD 0.13, 95% CI 0.02 to 0.25) at 4 weeks (Table 3 and Additional file 1: Figure S3–S5).

**Table 3:** Comparison of secondary outcomes between treatment groups^b^

Secondary outcomes measure^a^	LPT group vs HPT group		LPT group vs WL group		HPT group vs WL group	
	Mean difference (95% CI)	P value	Mean difference (95% CI)	P value	Mean difference (95% CI)	P value
WOMAC pain						
Baseline	–	–	–	–	–	–
4 weeks	−0.01 (−1.06 to 1.03)	0.98	−2.28 (−3.32 to −1.25)	< 0.001	−2.27 (−1.31 to −1.23)	< 0.001
8 weeks	−0.14 (−1.19 to 0.91)	0.80	−2.63 (−3.68 to −1.59)	< 0.001	−2.49 (−3.53 to −1.45)	< 0.001
12 weeks	0.45 (−0.60 to 1.51)	0.40	−2.16 (−3.20 to −1.11)	< 0.001	−2.61 (−3.66 to −1.57)	< 0.001
16 weeks	0.26 (−0.79 to 1.31)	0.63	−2.47 (−3.51 to −1.43)	< 0.001	−2.73 (−3.77 to −1.69)	< 0.001
WOMAC stiffness						
Baseline	–	–	–	–	–	–
4 weeks	−0.16 (−0.67 to 0.34)	0.52	−0.93 (−1.42 to −0.43)	< 0.001	−0.76 (−1.26 to −0.26)	0.003
8 weeks	−0.16 (−0.66 to 0.35)	0.54	−0.81 (−1.31 to −0.30)	0.002	−0.65 (−1.15 to −0.15)	0.01
12 weeks	−0.13 (−0.64 to 0.38)	0.61	−0.85 (−1.35 to −0.35)	0.001	−0.72 (−1.22 to −0.21)	0.005
16 weeks	−0.06 (−0.57 to 0.44)	0.80	−0.71 (−1.22 to −0.21)	0.005	−0.65 (−1.15 to −0.15)	0.01
WOMAC function						
Baseline	–	–	–	–	–	–
4 weeks	1.38 (−2.12 to 4.87)	0.44	−7.56 (−11.03 to −4.10)	< 0.001	−8.94 (−12.04 to −5.48)	< 0.001
8 weeks	0.48 (−3.03 to 4.00)	0.79	−7.45 (−10.93 to −3.96)	< 0.001	−7.93 (−11.41 to −4.45)	< 0.001
12 weeks	2.63 (−0.89 to 6.16)	0.14	−6.02 (−9.51 to −2.52)	< 0.001	−8.65 (−12.14 to −5.17)	< 0.001
16 weeks	2.32 (−1.18 to 5.83)	0.19	−7.22 (−10.70 to −3.74)	< 0.001	−9.55 (−13.02 to −6.08)	< 0.001
SF-12 PCS						
Baseline	–	–	–	–	–	–
4 weeks	1.09 (−0.16 to 2.34)	0.09	2.75 (1.51 to 3.99)	< 0.001	1.66 (0.42 to 2.90)	0.008
8 weeks	−0.47 (−1.74 to 0.79)	0.47	1.55 (0.30 to 2.81)	0.015	2.02 (0.77 to 3.27)	0.002
12 weeks	0.62 (−0.65 to 1.89)	0.34	1.93 (0.67 to 3.18)	0.003	1.30 (0.05 to 2.56)	0.04
16 weeks	0.10 (−1.15 to 1.36)	0.87	1.97 (0.72 to 3.21)	0.002	1.86 (0.62 to 3.11)	0.003
SF-12 MCS						
Baseline	–	–	–	–	–	–
4 weeks	−0.12 (−1.50 to 1.26)	0.86	−0.22 (−1.58 to 1.14)	0.75	−0.10 (−1.46 to 1.26)	0.88
8 weeks	0.15 (−1.24 to 1.54)	0.83	0.93 (−0.45 to 2.30)	0.19	0.78 (−0.60 to 2.15)	0.27
12 weeks	−0.50 (−1.89 to 0.90)	0.48	−0.17 (−1.55 to 1.22)	0.81	0.33 (−1.04 to 1.71)	0.64
16 weeks	−0.33 (−1.71 to 1.06)	0.64	0.56 (−0.81 to 1.93)	0.42	0.89 (−0.48 to 2.26)	0.2
Knee flexion ROM						
Baseline	–	–	–	–	–	–
4 weeks	0.79 (−0.97 to 2.54)	0.38	4.33 (2.59 to 6.08)	< 0.001	3.55 (1.81 to 5.29)	< 0.001
8 weeks	1.03 (−0.77 to 2.83)	0.26	3.32 (1.53 to 5.11)	< 0.001	2.30 (0.51 to 4.08)	0.01
12 weeks	0.88 (−0.99 to 2.74)	0.36	1.05 (−0.80 to 2.90	0.24	0.17 (−1.66 to 2.01)	0.85
16 weeks	0.53 (−1.36 to 2.42)	0.58	1.79 (−0.08 to 3.66)	0.06	1.25 (−0.60 to 3.11)	0.19
Knee extension ROM						
Baseline	–	–	–	–	–	–
4 weeks	0.13 (0.02 to 0.25)	0.02	0.06 (−0.06 to 0.18)	0.32	−0.07 (−0.19 to 0.04)	0.21
8 weeks	0.02 (−0.10 to 0.14)	0.79	0.10 (−0.02 to 0.21)	0.12	0.08 (−0.04 to 0.20)	0.2
12 weeks	0.05 (−0.08 to 0.17)	0.48	0.13 (0.01 to 0.25)	0.04	0.08 (−0.04 to 0.21)	0.17
16 weeks	0.03 (−0.10 to 0.15)	0.68	0.10 (−0.02 to 0.23)	0.1	0.08 (−0.05 to 0.20)	0.22
Knee internal rotation ROM						
Baseline	–	–	–	–	–	–
4 weeks	0.44 (−0.73 to 1.62)	0.46	1.28 (0.12 to 2.45)	0.03	0.84 (−0.33 to 2.00)	0.16
8 weeks	0.09 (−1.12 to 1.30)	0.89	1.61 (0.41 to 2.81)	0.009	1.52 (0.32 to 2.72)	0.01
12 weeks	0.14 (−1.12 to 1.39)	0.83	1.89 (0.65 to 3.12)	0.003	1.75 (0.52 to 2.98)	0.005
16 weeks	0.24 (−1.03 to 1.51)	0.71	0.52 (−0.74 to 1.77)	0.42	0.28 (−0.96 to 1.53)	0.66
Knee external rotation ROM						
Baseline	–		–		–	–
4 weeks	−0.42 (−1.63 to 0.80)	0.50	0.88 (−0.33 to 2.08)	0.15	1.30 (0.09 to 2.50)	0.03
8 weeks	0.34 (−0.90 to 1.59)	0.59	1.49 (0.16 to 2.63)	0.03	1.05 (−0.18 to 2.29)	0.09
12 weeks	0.43 (−0.86 to 1.71)	0.52	0.56 (−0.72 to 1.83)	0.39	0.13 (−1.13 to 1.40)	0.84
16 weeks	−0.44 (−1.74 to 0.87)	0.51	0.70 (−0.59 to 2.00)	0.28	1.14 (−0.14 to 2.42)	0.08

*LPT* lower pain threshold, *HPT* higher pain threshold, *WL* waiting-list

^a^Missing data not imputed for secondary outcomes analyses

^b^Adjusted analysis was performed using a liner mixed model with baseline value as covariate, treatment, visit, and interaction between treatment and visit as fixed effects, sites and individuals as random effects

At week 16, patients experienced greater improvements in WOMAC pain (MD −2.47, 95% CI −3.51 to -1.43), WOMAC stiffness (MD −0.71, 95% CI −1.22 to −0.21), WOMAC function (MD −7.22, 95% CI −10.70 to −3.74), and SF-12 PCS (MD 1.97, 95% CI 0.72 to 3.21) in the LPT group as compared to waiting-list group, but no significant differences were found in the improvement of SF-12 MCS, knee flexion ROM, knee extension ROM, knee internal rotation ROM and knee external rotation ROM (Table 3).

However, patients had greater improvements in knee flexion ROM in LPT over waiting-list group at 4 weeks (MD 4.33, 95% CI 2.59 to 6.08) and 8 weeks (MD 3.32, 95% CI 1.53 to 5.11). They also experienced greater improvements in knee internal rotation ROM at 12 weeks (MD 1.89, 95% CI 0.65 to 3.12) (Table 3).

Patients in the HPT group also experienced greater improvements in WOMAC pain (MD −2.73, 95% CI −3.77 to −1.69), WOMAC stiffness (MD −0.65, 95% CI −1.15 to −0.15), WOMAC function (MD −9.55, 95% CI −13.02 to −6.08), and SF-12 PCS (MD 1.86, 95% CI 0.62 to 3.11) compared with waiting-list group at 16 weeks (Table 3).

### Adverse events

Six patients (1.4%) reported acupuncture-related adverse events in the two acupuncture groups during the trial. Two patients in the LPT group experienced of a tingling sensation at acupoints after needles removal, and one had bruising in the area where the needle was inserted. Two patients from the HPT group had subcutaneous hemorrhage in the needle insertion area, and one had a fear about needles after receiving acupuncture treatment. All acupuncture-related adverse events were reported as mild, and resolved spontaneously during the study period.

## Discussion

In this multicenter RCT, administration of acupuncture at lower PT acupoints versus higher PT acupoints showed similar effects in reducing WOMAC total score at 16 weeks, and both were more effective than a waiting-list control. The incidence of acupuncture-related adverse events was low, and all events were reported as mild.

The analgesia of manual acupuncture is mainly achieved by stimulating somatic sensory functions of nervous system through *de qi* (i.e., producing a sensation of soreness, numbness, heaviness and distention) [35]. Central nervous system integrates the afferent sensory impulses from needling point—mainly C-type fibers—and pain area, releasing analgesic substances (e.g., opioid peptides) and activating the descending inhibitory system to relieve pain [35, 36]. The polymodal receptors of C-type fibers at acupoints are sensitive (i.e., with lower threshold) and easily activated by acupuncture, which in turn lead to these receptors insensitive by acupuncture stimulation and thus the analgesia effects is achieved by reducing the afferent pain sensation [24, 37]. Clinical studies have suggested that acupuncture can indeed increase the PT of needling point in patients with chronic musculoskeletal pain [22, 23]. This indicated that, as for patients with KOA, the polymodal-type receptors might be activated more easily when acupuncture is applied at acupoints with lower PT, resulting in activating endogenous antinociception system more effectively.

Our study showed that acupuncture at acupoints with lower versus higher PT had similar effects in KOA patients. In our trial, *de qi* was required in both LPT and HPT groups. As *de qi* is one of the most important factors affecting the therapeutic effect of manual acupuncture [38], and acupuncture with *de qi* could reach stimulus intensity threshold and activate the polymodal receptors to inhibit nociceptive neurons [39, 40], thus no differential effect was found between these two groups. In addition, we measured PT of 13 meridian acupoints and *ashi* points around knee, and the top five high frequency acupoints selected in HPT group, such as Dubi (ST35), Neixiyan (EX-LE4), Zusanli (ST36) and Heding (EX-LE2), were highly consistent with the most commonly recommended local acupoints [41]. We speculate that the effects of acupuncture at acupoints with lower PT selected by objective measurement might be similar to that of traditional empirical acupoints. Therefore, PT might be a quantifiable approach for acupoints, but selection of acupoints around knee by PT might not be suitable for patients with KOA in acupuncture clinical practice.

The effect of acupuncture on WOMAC total score at 16 weeks in this trial was clinically important with 44.62% (23.13/51.84) mean improvement from baseline in LPT group and 49.17% (27.38/55.69) in HPT group, which exceeded than the minimal clinically important difference (MCID) of WOMAC total score defined as 16% [42]. Our findings were consistent with a previous clinical trial [13], which showed a 47.0% (23.9/50.8) WOMAC total score reduction at 8 weeks of acupuncture treatment. The reduction in WOMAC total score of acupuncture treatment in KOA patients was similar to that of intraarticular injections of glucocorticoid up to three times a year (48.71%, 55.8/108.8) [43], and was significantly greater than that of 9-month stepped exercise program (11.58%, 5.5/47.5) [44]. Our study showed that acupuncture could significantly improve symptoms and function of KOA as measured by WOMAC, therefore, acupuncture could be recommended as a non-pharmacological treatment for patients with KOA.

In this study, all acupuncture-related adverse events were mild, and patients were fully recovered during the study period. Evidence from several large surveys have suggested that acupuncture is a relatively safe therapy [45, 46]. Clinical guidelines varied widely regarding acupuncture recommendations for KOA. The American College of Rheumatology conditionally recommends acupuncture for people with KOA [6], while the American Academy of Orthopaedic Surgeons rated acupuncture as “strongly not recommended” for KOA [47]. Findings from this study support the acupuncture recommendation for patients with KOA in clinical guidelines.

### Strengths and limitations

In our study, the identification, measurement and selection of acupoints were performed strictly based on a standardized protocol. The electronic Von Frey was used to measure the PT, which provided reliable and accurate assessment. The other advantages to this study included strictly concealed central randomization, blinded outcome evaluation, standardized and validated questionnaires, qualified and experienced acupuncturists, an independent data monitoring committee, and high follow-up rates.

Our study also has limitations. First, due to the nature of acupuncture intervention, it was not possible to blind patients and acupuncturists to treatment. However, patients in LPT group and HPT group were informed receiving the same acupuncture treatment. Second, patients in this trial received four weeks of acupuncture treatment, while all outcome measures were conducted at baseline (pre-treatment), 4 (post-treatment), 8, 12 and 16 weeks, therapeutic effect evaluation during the acupuncture treatment period (e.g., 2 weeks) might be missing, especially in the course of dynamic changes of PT. Third, due to time and labor factors, we measured PT of acupoints only once for each patient, and applied acupuncture at the same acupoints in the following 12 sessions, which did not ensure that the five acupoints selected with lower or higher PT originally were still the desired acupoints in the following treatment. Fourth, this study did not establish a sham acupuncture group to investigate the placebo effect of acupuncture, however, we added a waiting-list group to control for regression to mean in the natural history of disease [48]. Finally, our findings cannot be generalized to patients with severe or late clinical stage of KOA.

## Conclusions

Our findings showed that administration of acupuncture at acupoints with lower versus higher PT had similar effects in patients with KOA. Our results confirmed the positive effects of acupuncture on pain, stiffness, physical function and quality of life of in patients with KOA.

## Supplementary Information

**Additional file1**: **Figure S1.** The anterior and posterior testing regions of knee. **Figure S2.** Subgroup analyses for the primary outcome comparing LPT group and HPT group. **Figure S3.** WOMAC pain (A), stiffness (B) and function (C) subscales over time during the study. **Figure S4.** SF-12 PCS (A) and MCS (B) scores over time during the study. **Figure S5.** The active knee ROMs over time during the study.

**Additional file 2:** **Table S1.** Co-interventions used among treatment groups.

## Data Availability

The protocol and the predefined data analysis plan of this trial are available on request. Statistical code: available from Dr. Jiali. Liu (email, tiedaobuliujiali@163.com). Data set: certain portions of the analytic data set are available to approved individuals through written agreements with the corresponding author.

## Abbreviations

PT: Pain threshold
LPT: Lower pain threshold
HPT: Higher pain threshold
KOA: Knee osteoarthritis
WOMAC: Western ontario and mcmaster universities osteoarthritis index
SF-12: 12-Item short form health survey
PCS: Physical component score
MCS: Mental component score
ROM: Knee range of motion
CAM: Complementary and alternative medical
RCT: Randomized clinical trial
DMC: Data monitoring committee
