# Supplementary material for: Effects of acupuncture at acupoints with lower versus higher pain threshold for knee osteoarthritis: a multicenter randomized controlled trial
Source: Chin Med. 2022 Jun 8;17:67. doi: 10.1186/s13020-022-00626-3 (PMC9175460; doi:10.1186/s13020-022-00626-3)
Supplement: Supplementary file 1 — Additional file1: Figure S1. The anterior and posterior testing regions of knee. Figure S2. Subgroup analyses for the primary outcome comparing LPT group and HPT group. Figure S3. WOMAC pain (A), stiffness (B) and function (C) subscales over time during the study. Figure S4. SF-12 PCS (A) and MCS (B) scores over time during the study. Figure S5. The active knee ROMs over time during the study. [file 13020_2022_626_MOESM1_ESM.pdf]

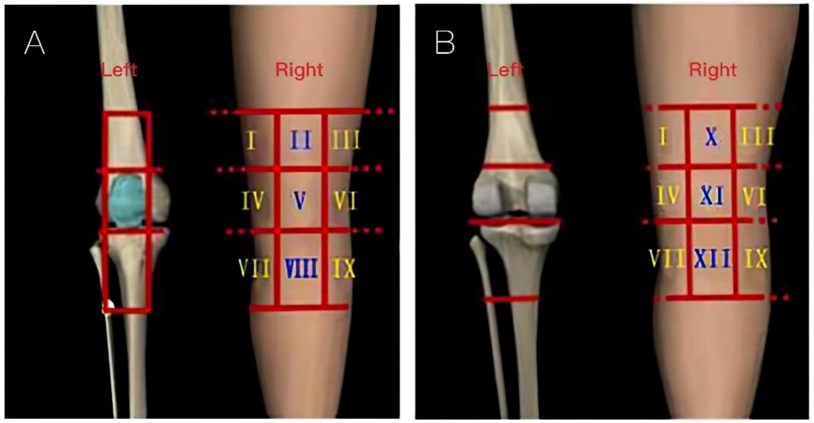

Figure S1. The anterior and posterior testing regions of knee

The anterior and posterior regions of knee were divided into 12 testing areas. The division was as follows: draw a line horizontally 7.6 cm above the patella (the upper boundary of the testing zones); draw two lines horizontally between the upper and low of patella; draw a line horizontally across Zusanli (ST36) (the low boundary of the testing zones); in the anterior region, draw two lines vertically on the inner and outer top corners of the basis patellae; in the posterior region, draw two lines which vertically trisect the popliteal area.

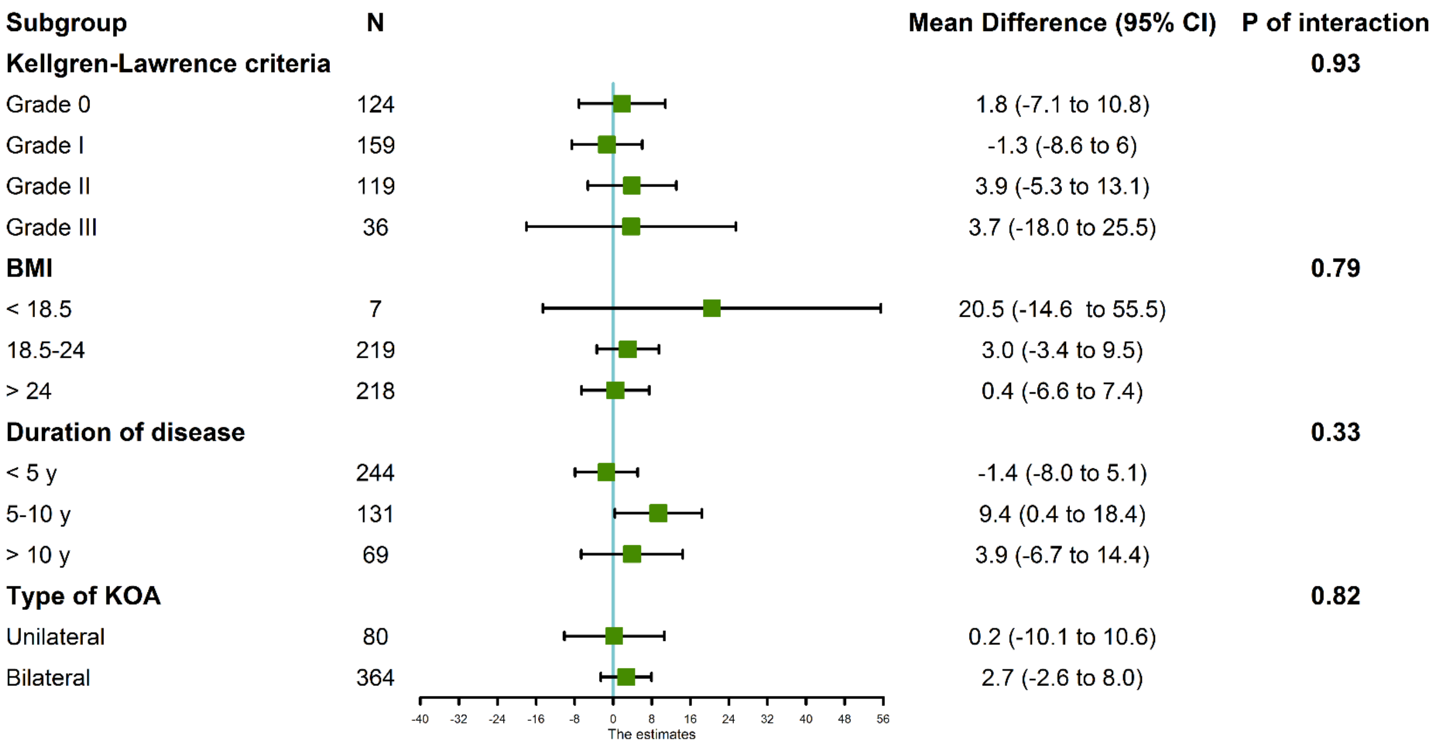

Figure S2. Subgroup analyses for the primary outcome comparing LPT group and HPT group

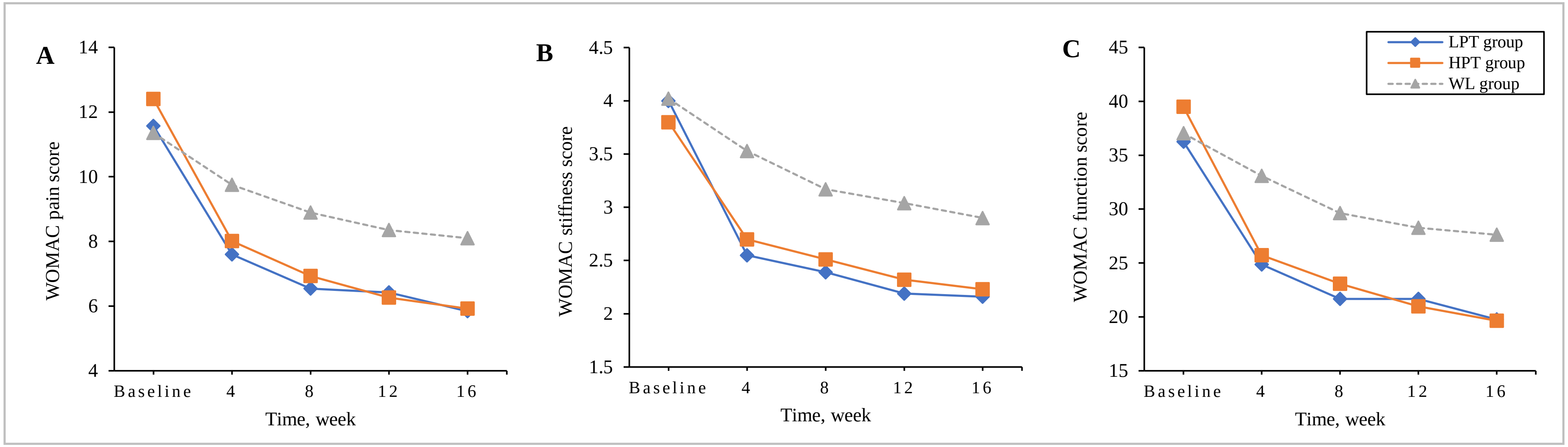

Figure S3. WOMAC pain (A), stiffness (B) and function (C) subscales over time during the study

LPT: lower pain threshold, HPT: higher pain threshold, WL: waiting-list.

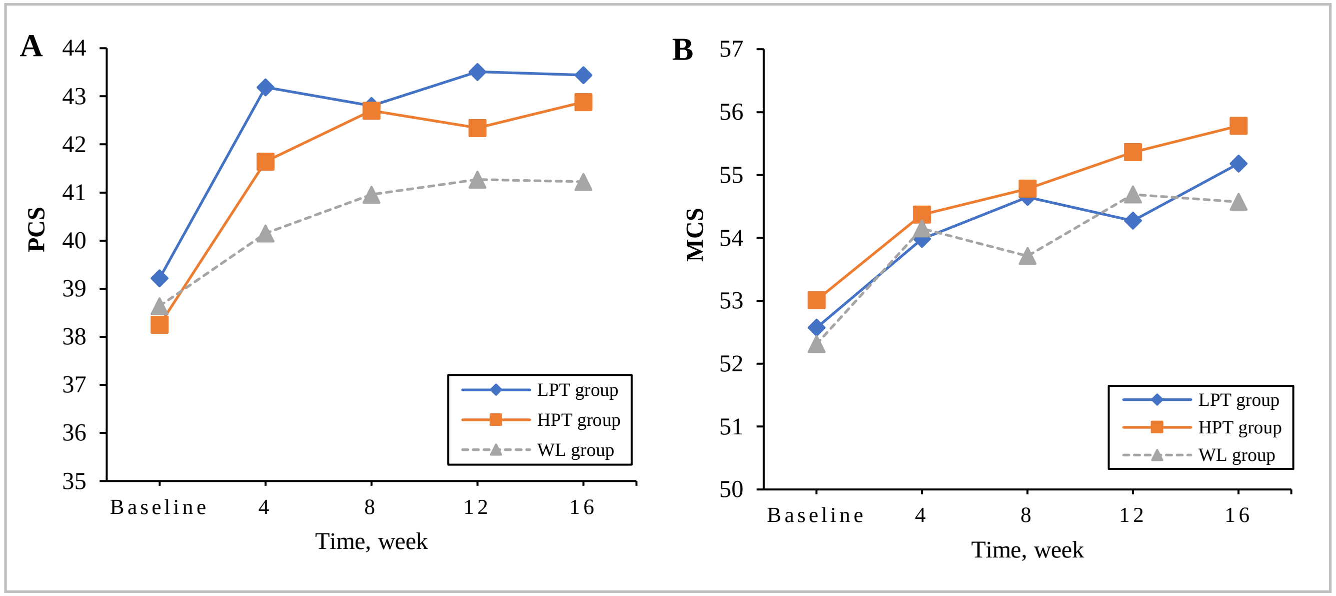

Figure S4. SF-12 PCS (A) and MCS (B) scores over time during the study.

LPT: lower pain threshold, HPT: higher pain threshold, WL: waiting-list.

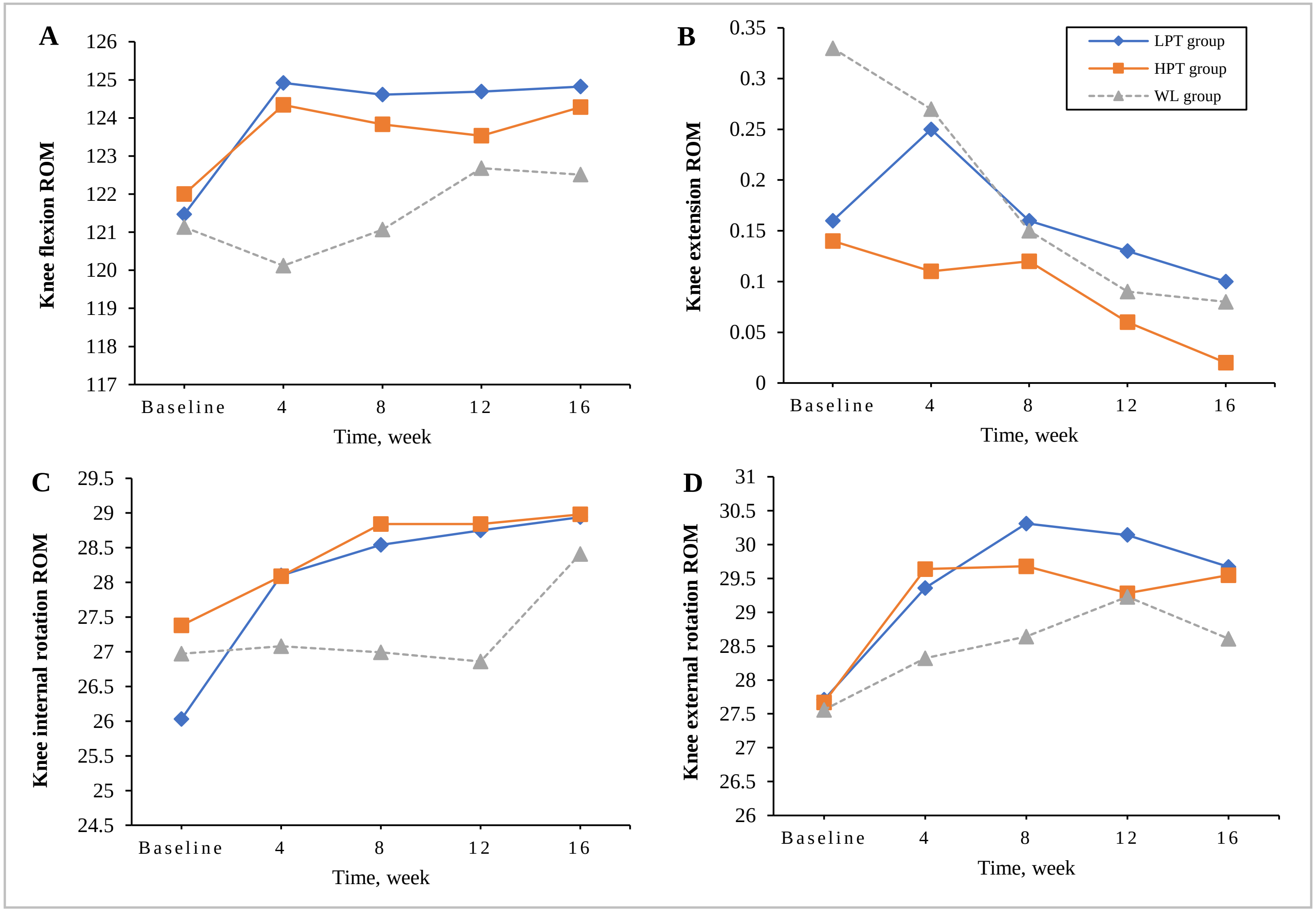

Figure S5. The active knee ROMs over time during the study.

LPT: lower pain threshold, HPT: higher pain threshold, WL: waiting-list.
