# Supplementary material for: Effects of acupuncture at acupoints with lower versus higher pain threshold for knee osteoarthritis: a multicenter randomized controlled trial
Source: Chin Med. 2022 Jun 8;17:67. doi: 10.1186/s13020-022-00626-3 (PMC9175460; doi:10.1186/s13020-022-00626-3)
Supplement: Supplementary file 2 — Additional file 2: Table S1. Co-interventions used among treatment groups. [file 13020_2022_626_MOESM2_ESM.pdf]

Table S1. Co-interventions used among treatment groups.

Co-interventions, n(%)	LPT group (n=222)	HPT group (n=222)	WL group (n=222)	All (n=666)
**Medication use during study**				
NSAIDs	5 (2.25)	3 (1.35)	5 (2.25)	13 (1.95)
Glucosamine	52 (23.42)	57 (25.68)	55 (24.77)	164 (24.62)
Diacerein	1 (0.45)	3 (1.35)	2 (0.9)	6 (0.9)
Vitamin D	4(1.8)	5(2.25)	6(2.7)	15(2.25)
Alendronate	2 (0.9)	0 (0)	0 (0)	2 (0.3)
Tizanidine hydrochloride	1 (0.45)	0 (0)	0 (0)	1 (0.15)
Sodium hyaluronate	1 (0.45)	0 (0)	0 (0)	1 (0.15)
**TCM use during study**				
Electromagnetic spectrum irradiation	12 (5.41)	7 (3.15)	8 (3.6)	27 (4.05)
Moxibustion	3 (1.35)	2 (0.9)	8 (3.6)	13 (1.95)
Massage	5 (2.25)	2 (0.9)	10 (4.5)	17 (2.55)
Medicinal liquor	8 (3.6)	6 (2.7)	6 (2.7)	20 (3)
Cupping	2 (0.9)	0 (0)	2 (0.9)	4 (0.6)

NSAIDs: nonsteroidal anti-inflammatory drugs; TCM: Traditional Chinese Medicine

LPT: lower pain threshold; HPT: higher pain threshold; WL: waiting-list.
